# LRRK2 Phosphorylates Tubulin-Associated Tau but Not the Free Molecule: LRRK2-Mediated Regulation of the Tau-Tubulin Association and Neurite Outgrowth

**DOI:** 10.1371/journal.pone.0030834

**Published:** 2012-01-27

**Authors:** Fumitaka Kawakami, Takatoshi Yabata, Etsuro Ohta, Tatsunori Maekawa, Naoki Shimada, Minori Suzuki, Hiroko Maruyama, Takafumi Ichikawa, Fumiya Obata

**Affiliations:** 1 Department of Biochemistry, Graduate School of Medical Sciences, Kitasato University, Sagamihara, Japan; 2 Department of Clinical Immunology, Graduate School of Medical Sciences, Kitasato University, Sagamihara, Japan; 3 Laboratory of Cytopathology, Graduate School of Medical Sciences, Kitasato University, Sagamihara, Japan; 4 Research and Development Center for Cell Design, Institute for Regenerative Medicine and Cell Design, School of Allied Health Sciences, Kitasato University, Sagamihara, Japan; Hertie Institute for Clinical Brain Research and German Center for Neurodegenerative Diseases, Germany

## Abstract

Leucine-rich repeat kinase 2 (LRRK2), a large protein kinase containing multi-functional domains, has been identified as the causal molecule for autosomal-dominant Parkinson's disease (PD). In the present study, we demonstrated for the first time that (i) LRRK2 interacts with tau in a tubulin-dependent manner; (ii) LRRK2 directly phosphorylates tubulin-associated tau, but not free tau; (iii) LRRK2 phosphorylates tau at Thr181 as one of the target sites; and (iv) The PD-associated LRRK2 mutations, G2019S and I2020T, elevated the degree of tau-phosphorylation. These results provide direct proof that tau is a physiological substrate for LRRK2. Furthermore, we revealed that LRRK2-mediated phosphorylation of tau reduces its tubulin-binding ability. Our results suggest that LRRK2 plays an important role as a physiological regulator for phosphorylation-mediated dissociation of tau from microtubules, which is an integral aspect of microtubule dynamics essential for neurite outgrowth and axonal transport.

## Introduction

Tau is a microtubule-associated protein found predominantly in the central nervous system and expressed mainly in neuronal axons [1]. Tau has six splicing isoforms, ranging in size from 352 to 441 amino acid residues [2]. The shortest tau isoform is expressed only in fetal brain, and the other five are expressed developmentally in the adult brain [3]. Tau drives neurite outgrowth by promoting the assembly of microtubules, which is critical for the establishment of neuronal cell polarity [4]. In Alzheimer's disease and other neurodegenerative diseases, such as frontotemporal dementia and parkinsonism linked to chromosome 17 (FTDP-17), tau becomes highly phosphorylated and forms a paired helical filament [3]. Hyperphosphorylated tau-based neurofibrillary lesions are the predominant brain pathology in these disorders, which are referred to collectively as “tauopathies” [5].

Leucine-rich repeat kinase 2 (LRRK2) is the causative molecule of familial Parkinson's disease (PD) [6], [7]. It is a 286-kDa protein containing an N-terminal leucine-rich repeat, a Ras of complex protein (ROC) GTPase domain, a C-terminal of the Roc region, a kinase domain, and a WD40 domain [8]. LRRK2 is widely expressed in many organs, such as the brain, heart, kidney, lung, and liver [9], [10]. It is also expressed in some immune cells [11], [12]. In the brain, LRRK2 is expressed in the cerebral cortex, medulla, cerebellum, spinal cord, putamen, and substantia nigra. Accumulating evidence [13] indicates that (i) wild-type LRRK2 exhibits protein kinase activity, and undergoes autophosphorylation; (ii) the kinase activity of LRRK2 has a strict requirement for binding of guanosine triphosphate (GTP), whereas intrinsic GTPase activity is exerted independently of the kinase activity; and (iii) the phosphorylation activity of LRRK2 is enhanced significantly by the G2019S mutation linked to the pathogenesis of PD. However, neither the physiological function of LRRK2 including the true substrate(s) nor the molecular mechanisms of neurodegeneration caused by LRRK2 mutations has yet been elucidated.

It has been reported that neurite length is reduced in LRRK2-deficient cultured mouse neurons [14]. In contrast, another study found that neurite length and branching were increased by LRRK2-knockdown or LRRK2-kinase inactivation and reduced by PD-associated LRRK2 mutations [15]. Furthermore, studies of the kinase-active mutant G2019S-LRRK2 have demonstrated common morphological changes in neurites, i.e., i) neurite shortening due to G2019S-LRRK2 expression in differentiated SH-SY5Y cells [16], [17], ii) shortened neurites of cultured neurons derived from G2019S-LRRK2 transgenic mice [18], and iii) markedly reduced neurite complexity of cultured dopaminergic neurons in the brains of aged G2019S-LRRK2 transgenic mice [19]. LRRK2 might regulate neuronal morphology through interaction with, and phosphorylation of, β-tubulin [14]. In addition, several observations such as increased or reduced phosphorylation of tau, mislocalization of tau, and phospho-tau-positive inclusions in neurons of animal models and patients with LRRK2 abnormality [14], [15], [20]–[23] strongly suggest that LRRK2 may modulate microtubule dynamics by controlling the phosphorylation status of tau. However, because no experimental evidence proving that LRRK2 directly phosphorylates tau has been reported, the contribution of LRRK2 to phosphorylation of tau is thought to be indirect, occurring via other kinases such as glycogen synthase kinase-3β (GSK-3β) and thousand-and-one amino acid kinase 3 (TAOK3) [15], [23], [24]. In the present study, we demonstrate that LRRK2 directly phosphorylates tau in the presence of tubulin and facilitates dissociation of tau from tubulin, thus indicating that LRRK2 is of considerable physiological importance in microtubules dynamics.

## Materials and Methods

### Chemicals

[γ-^32^P]ATP (3000 Ci/mmol) was obtained from Perkin Elmer Inc. (Massachusetts, USA); dithiothreitol (DTT) was from Wako Pure Chemical (Osaka, Japan); recombinant N-terminal glutathione S-transferase (GST)-tagged LRRK2 (GST–LRRK2, aa 970–2527; wild-type, R1441C mutant, G2019S mutant, and I2020T mutant) were from Invitrogen (San Diego, USA); recombinant tau protein 441 (tau, aa 1–441) was from Signal Chem Pharmaceuticals (Richmond, Canada); purified porcine tubulin was from Cytoskeleton (Denver, USA); and bovine myelin basic protein (MBP) was from Sigma-Aldrich (Missouri, USA).

### Antibodies

Anti-GST was obtained from Advanced Targeting Systems (San Diego, USA); horseradish peroxidase (HRP)-conjugated anti-V5 and anti-V5 antibody-conjugated agarose beads were from Invitrogen (Camarillo, USA); rabbit anti-LRRK2 monoclonal antibody MJFF2 (c41-2) was from Epitomics (Burlingame, USA); anti-human tau (HT7), anti-phosphorylated Thr residues of tau, AT270 (Thr181), AT8 (Thr205), AT100 (Thr212), and AT180 (Thr231) were from Thermo Fisher Scientific (Fremont, USA); anti-beta tubulin for Western analysis was from Abcam (Cambridge, UK); and anti-beta-III tubulin for immunofluorescent staining was from R&D Systems (Minneapolis, USA). HRP-conjugated goat anti-mouse IgG and HRP donkey anti-rabbit IgG were from Biolegend (San Diego, USA) and Alexa Fluor 555 goat anti-mouse IgG was from Cell Signaling (Danvers, USA).

### Cell culture and Immunoprecipitation

A clone (WT4-D33) of the human neuroblastoma cell line SH-SY5Y, stably expressing LRRK2 with a C-terminal V5-tag and a vector control clone (NEO) expressing only the neomycin resistant gene have been described previously [25]. The cells were cultured in Dulbecco's modified Eagle medium (DMEM) nutrient mixture F-12 HAM (Sigma) supplemented with 10% FCS and antibiotics at 37°C and 5% CO_2_ in a humidified atmosphere, as described previously [25]–[27]. The cells were lysed in lysis buffer [10 mM Tris–HCl, pH 7.4, 150 mM NaCl, 1 mM EDTA, 1 mM EGTA, 1% NP-40, protease and phosphatase inhibitor cocktail (Roche, Mannheim, Germany)]. The cell lysates were incubated with anti-V5 antibody-conjugated or normal rabbit IgG-conjugated agarose beads overnight at 4°C. The beads were then washed three times with lysis buffer, and precipitated proteins were detected by Western blotting using the antibodies indicated in the figure legends.

### *In vitro* GST pull-down assay

Recombinant GST-LRRK2 or GST alone was mixed with porcine tubulin and incubated with glutathione agarose beads overnight at 4°C in 100 µL of TNE buffer [50 mM Tris-HCl (pH 7.4), 150 mM NaCl, 1% (v/v) NP-40, 1 mM EDTA]. In another experiment, recombinant tau was mixed with GST-LRRK2 or GST alone and incubated with glutathione agarose beads in the presence or absence of tubulin in 100 µL of TNE buffer overnight at 4°C. After centrifugation, the supernatant was removed, and the beads were washed three times with TNE buffer. The bound proteins were eluted from the beads by boiling in 50 µL of SDS-PAGE sample buffer. The GST-LRRK2 (or GST alone), tau and tubulin in the eluted samples were detected by Western blotting.

### *In vitro* kinase assay for LRRK2

Recombinant tau was used as a substrate for GST-LRRK2. Briefly, 2 µg of tau was incubated with 50 ng of GST-LRRK2 (either wild-type, R1441C mutant, G2019S mutant, or I2020T mutant) in 25 µL of standard reaction mixture [40 mM Tris-HCl (pH 7.6), 2 mM DTT, 10 mM Mg^2+^, 3 µCi of [γ-^32^P]ATP] in the presence or absence of tubulin (1 µg). After incubation for 30 min at 30°C, the reaction was stopped by adding SDS-PAGE sample buffer and boiling. The ^32^P-labeled proteins in the reaction mixture were detected by autoradiography followed by SDS-PAGE, as described previously [27]–[29].

### Phosphoamino acid and phosphopeptide analysis of tau

Detection of phosphoamino acids and phosphopeptides of ^32^P-labeled tau was performed by two-dimensional TLC as described previously [28], [29].

### Phosphorylation of tau and neurite outgrowth in LRRK2-knockdown cells

For knockdown of LRRK2 expressed by the SH-SY5Y clone WT4-D33 and a vector control clone NEO, cells were transfected with 25 mer of Stealth® RNAi for LRRK2 (5′-GAGCUGCUCCUUUGAAGAUACUAAA-3′; Invitrogen) or with an RNAi-control with the scrambled sequence using FuGENE®HD Transfection Reagent (Roche), as described previously [27]. After 48 h of transfection, cell lysates were prepared and subjected to Western analysis with antibodies against V5-tag, LRRK2 (MJFF2), phosphorylated tau (Thr181), and non-phosphorylated tau. For analysis of neurite outgrowth, WT4-D33 and NEO were first treated with 10 µM all-trans retinoic acid for 72 h, transfected with the LRRK2-specific RNAi or RNAi-control, cultured for a further 72 h, and subjected to immunofluorescence staining with anti-β III tubulin. The length of neurites was analyzed using NIH ImageJ software (NeuronJ).

## Results

### Tubulin-dependent interaction of LRRK2 with tau

**Figure 1:**
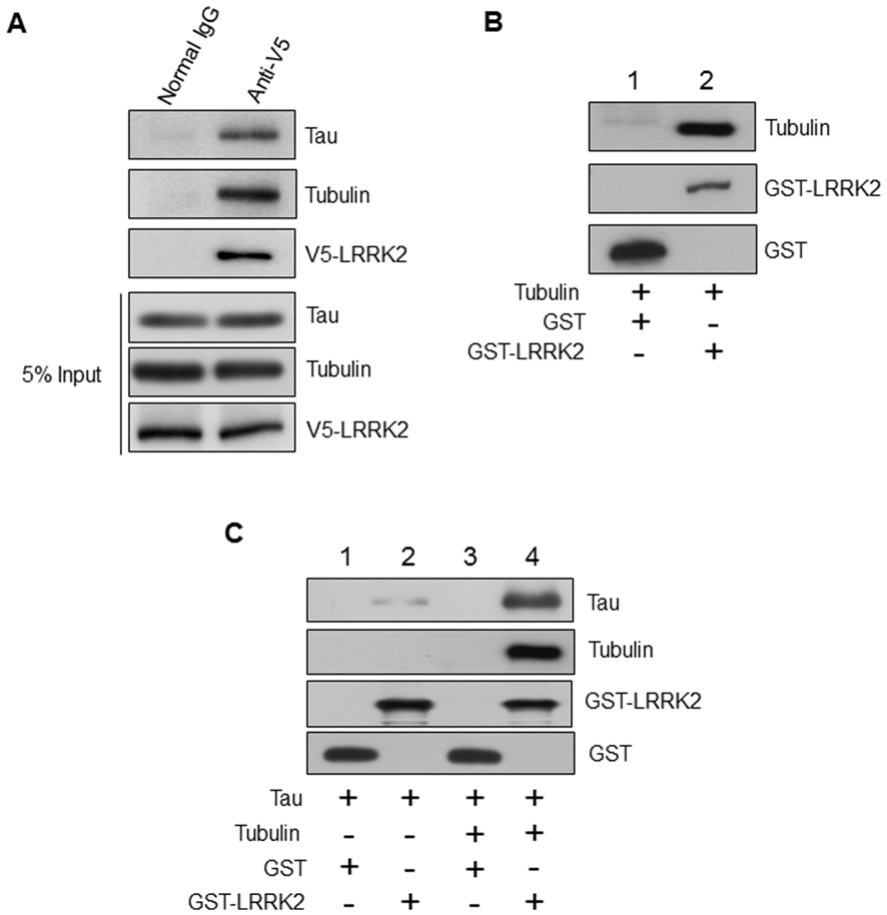
Tubulin-dependent interaction of LRRK2 with tau. (A) V5-LRRK2 was immunoprecipitated with anti-V5-agarose beads from the V5-LRRK2-stably-expressing SH-SY5Y clone, WT4-D33. Normal rabbit IgG-conjugated agarose beads were used as a control. The immunoprecipitates were separated by SDS-PAGE, transferred to PVDF, and analyzed using antibodies against V5-tag, tau, and tubulin. (B) GST (lane 1) and GST-LRRK2 (lane 2) were incubated with porcine tubulin, and a pull-down assay using glutathione-agarose beads was performed. Precipitated proteins were detected by Western analysis using anti-GST and anti-tubulin antibodies. (C) GST and GST-LRRK2 were incubated with recombinant tau in the absence (lane 1 and 2) or presence (lane 3 and 4) of porcine tubulin, and a pull-down assay using glutathione-agarose beads was performed. Precipitated proteins were detected by Western analysis using the indicated antibodies.

To examine the possibility that LRRK2 is able to interact with tau in human neural cells, we first performed a co-immunoprecipitation assay. A cell lysate of a SH-SY5Y clone (WT4-D33 [25]) stably overexpressing V5-tagged full length LRRK2 was incubated with anti-V5 antibody-conjugated agarose beads. The precipitated proteins were then detected by Western blotting using specific antibodies against V5-tag, tau and tubulin. We found that both tau and tubulin were co-precipitated with LRRK2 (Fig. 1A).

To elucidate the relationships among the three interacting molecules (LRRK2, tubulin, and tau), we performed an *in vitro* pull-down assay using recombinant proteins. First, recombinant GST-LRRK2 was mixed with porcine tubulin. We confirmed the presence of specific and direct binding between LRRK2 and tubulin, consistent with the report by Gillardon [14] (Fig. 1B). Second, recombinant tau was mixed with GST-LRRK2 in the presence or absence of tubulin, and then incubated with glutathione agarose beads. In the presence of tubulin, a large amount of tau was co-precipitated with LRRK2, whereas in the absence of tubulin little tau was co-precipitated (Fig. 1C, lane 4 vs lane 2). These results suggested that LRRK2 was able to bind tubulin-associated tau but not the free tau molecules.

### LRRK2 phosphorylates tubulin-associated tau but not free tau

**Figure 2:**
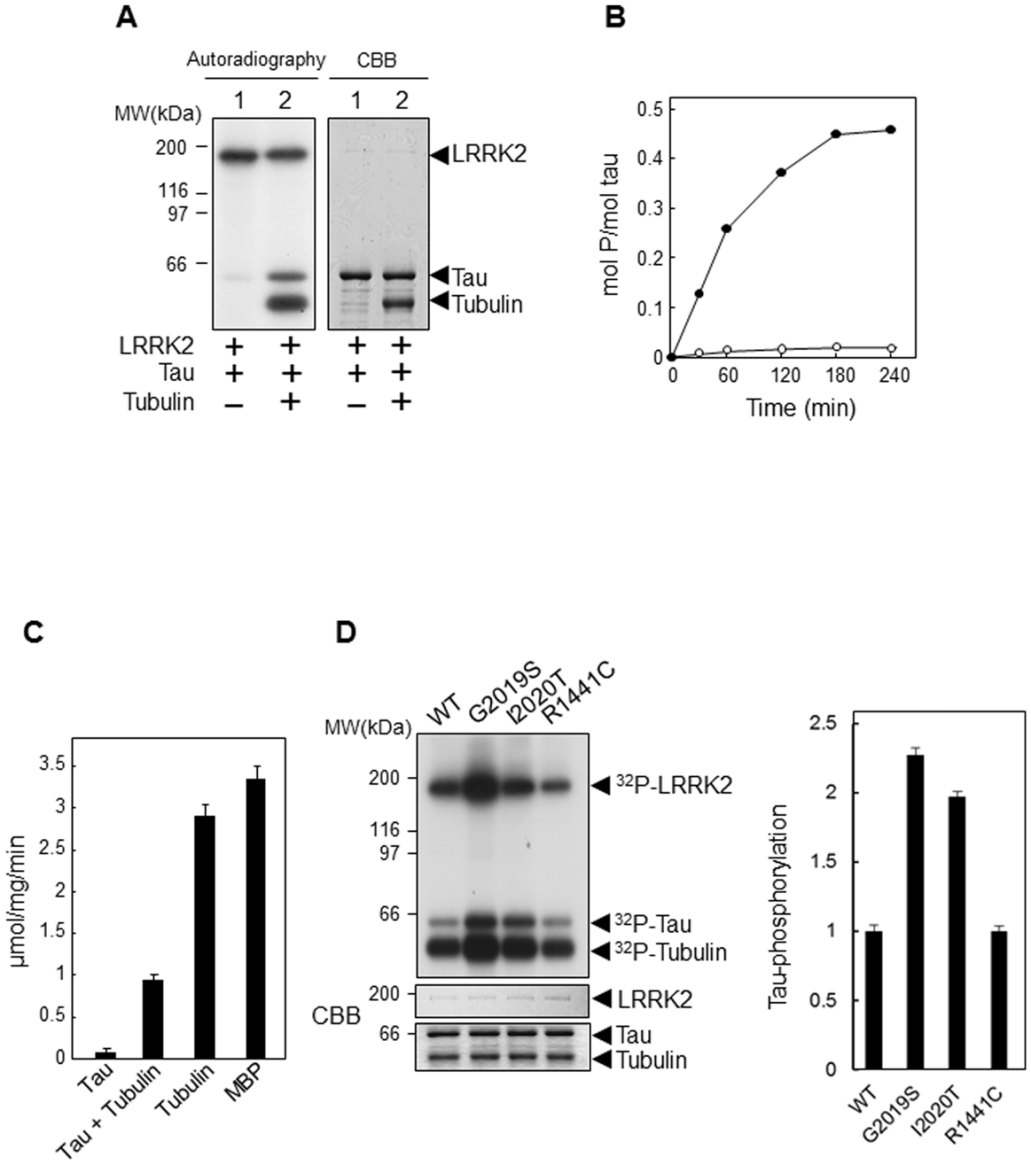
LRRK2-mediated phosphorylation of tau in the presence of tubulin. (A) Recombinant tau (2 µg) was incubated with GST-LRRK2 (50 ng each) and 3 µCi of [γ-^32^P]ATP in the absence (lane 1) or presence (lane 2) of purified porcine tubulin (1 µg) for 30 min at 30°C. ^32^P-Labeled proteins in the reaction mixture were detected by autoradiography following SDS-PAGE. A Coomassie Brilliant Blue (CBB) post-stained gel image is shown in the right panel to indicate the identity and amount of LRRK2 and tau in the two lanes. (B) The kinetics of tau phosphorylation by LRRK2 was measured after incubation for indicated periods at 30°C. The radioactivity of ^32^P-labeled tau in the presence (•) or absence (○) of tubulin was determined using a liquid scintillation counter. (C) Equal amounts (2 µg) of tau, tubulin, and MBP were incubated with GST-LRRK2 (50 ng each) and 3 µCi of [γ-^32^P]ATP for 30 min at 30°C. The radioactivity of ^32^P-labeled proteins was determined using a liquid scintillation counter. (D) Left: Tau (2 µg) was incubated with GST-LRRK2 of the wild-type (WT), G2019S, I2020T, or R1441C mutants and 3 µCi of [γ-^32^P]ATP in the presence of tubulin (1 µg) for 30 min at 30°C. The ^32^P-labeled proteins in the reaction mixture were detected by autoradiography following SDS-PAGE. CBB post-stained gel images are shown in the bottom panel to indicate the identity and amount of LRRK2, tau, and tubulin in the lanes. Right: Graphical representation of the rates of tau phosphorylation by WT, G2019S, I2020T, and R1441C LRRK2. Phosphorylation rates relative to WT-LRRK2 are shown.

We then investigated whether tubulin affects the phosphorylation of tau by LRRK2. Recombinant tau was incubated with GST-LRRK2 and [γ-^32^P]ATP in the presence or absence of tubulin. We found that LRRK2 markedly phosphorylated tau in the presence of tubulin, whereas it phosphorylated tau only very slightly in the absence of tubulin (Fig. 2A). These results indicated that LRRK2 directly phosphorylated tubulin-associated tau, but not tubulin-unassociated free tau.

To determine the stoichiometry of tau phosphorylation by LRRK2, incorporation of ^32^P into tau was measured after incubation with LRRK2, tubulin, and radiolabeled ATP. It was found that about 0.5 mole of phosphate was incorporated into 1 mole of tau (Fig. 2B). This low phosphorylation level was ascribed to the fact that only tubulin-associated tau, and not free tau, is phosphorylated by LRRK2. The kinase activity of LRRK2 toward tubulin-associated tau was approximately three times lower than that toward known excellent substrates such as tubulin or MBP under our experimental conditions (Fig. 2C). We further analyzed the tau-phosphorylation activity of LRRK2 with three PD-associated mutations: R1441C in the ROC domain, G2019S in the kinase domain, and I2020T in the kinase domain. We found that G2019S and I2020T, but not the R1441C mutant LRRK2, exerted increased tau-phosphorylation activity in comparison with the wild-type LRRK2 (Fig. 2D). These results indicate that the phosphorylation of tau is indeed attributable to the kinase activity of LRRK2, which is influenced by disease-associated mutations.

### Identification of phosphorylation site for LRRK2 in tau

**Figure 3:**
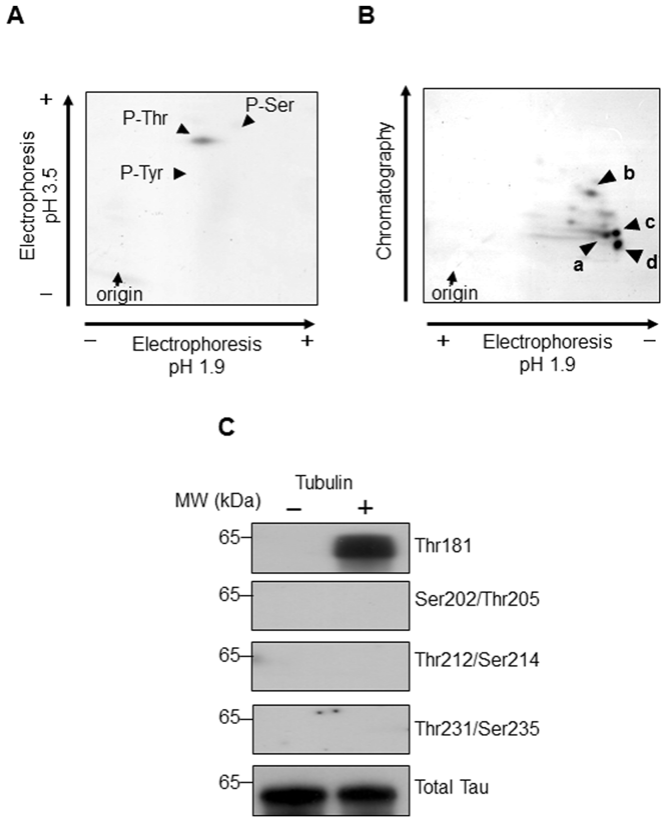
Identification of phosphorylation site for LRRK2 in tau. (A) ^32^P-labeled phosphoamino acids of tau fully phosphorylated by GST-LRRK2 in the presence of tubulin were analyzed by two-dimensional thin-layer cellulose (TLC) electrophoresis, as described in Materials and Methods. (B) ^32^P-labeled tau was separated by SDS-PAGE and digested with trypsin in the gel. The resulting polypeptides were separated by TLC electrophoresis (first dimension) and chromatography (second dimension). The phosphopeptides were loaded at the position indicated as the origin. (C) Tau was incubated with GST-LRRK2 and 100 µM non-radiolabeled ATP in the presence or absence of tubulin at 30°C for 180 min. The samples were then analyzed by Western blotting using antibodies specific to the phosphorylated Thr residues of tau.

Next, we performed phosphoamino acid analysis after full phosphorylation of tau by GST-LRRK2 using [γ-^32^P]ATP. Only the ^32^P-labeled Thr residue was detected when tau was incubated with GST-LRRK2 and tubulin, and no phosphorylation of the Ser or Tyr residue was observed (Fig. 3A). Phosphopeptide mapping of ^32^P-labeled tau revealed four major phosphorylated fragments (a–d) (Fig. 3B). These findings suggested that at least four potential phosphorylation sites (Thr only) of tau are predominantly phosphorylated by LRRK2 in the presence of tubulin. To identify the site of tau phosphorylation by LRRK2, we performed Western analysis using antibodies specific for each of the phospho-Thr residues, i.e., AT270 (Thr181), AT8 (Thr205), AT100 (Thr212), and AT180 (Thr231), which are the sites known to be frequently phosphorylated. We found that Thr181 was phosphorylated by LRRK2 only in the presence of tubulin (Fig. 3C). These results indicated that Thr181 is one of the phosphorylation target sites for LRRK2. In addition, tau may contain three unidentified potential LRRK2-target sites, because four major phosphorylated fragments were detected by phosphopeptide mapping.

### Effect of overexpression and knockdown of LRRK2 on phosphorylation of tau (Thr181) and neurite outgrowth in neuronal cells

**Figure 4:**
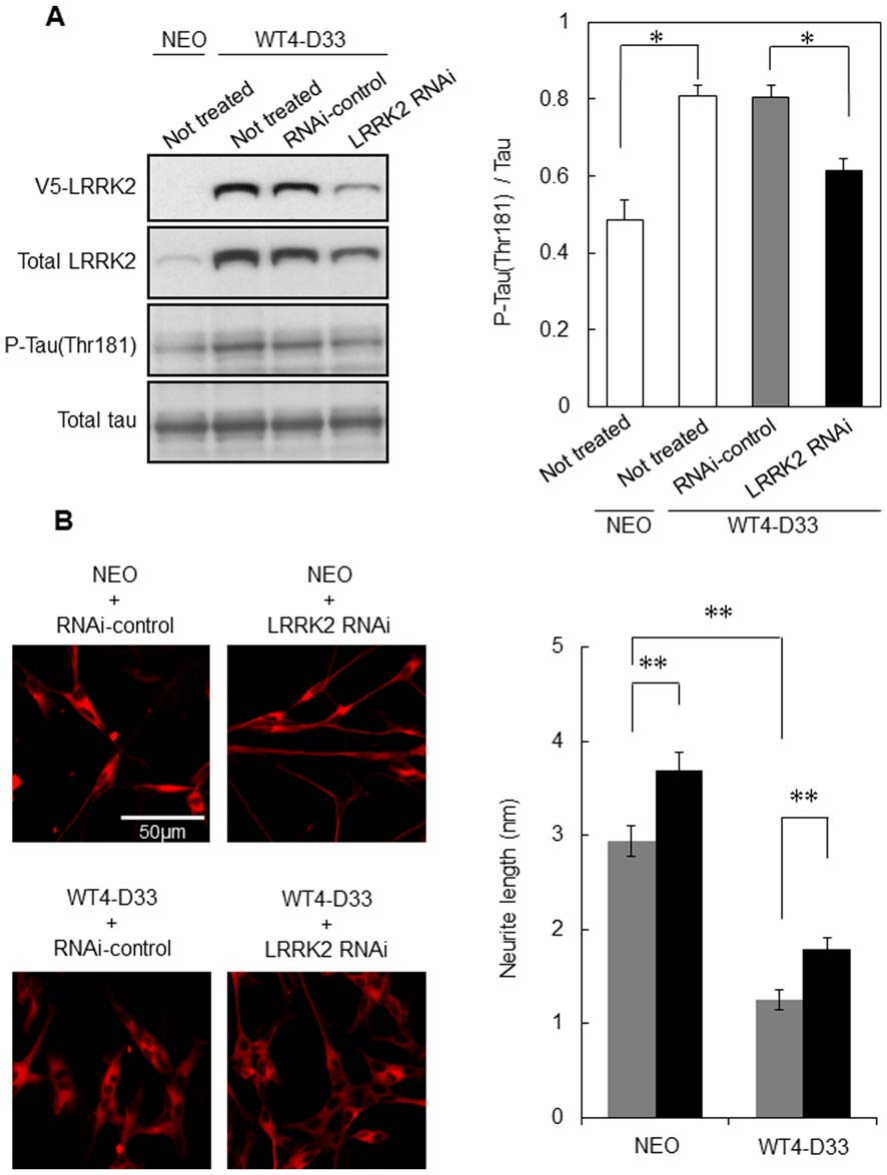
Effect of overexpression and knockdown of LRRK2 on phosphorylation of tau (Thr181) in neuronal cells and their neurite outgrowth. (A) Left: A SH-SY5Y clone (WT4-D33) expressing V5-tagged wild-type LRRK2 was transfected with either the LRRK2-specific RNAi or the RNAi control. After 48 h of transfection, cell lysates were prepared and subjected to Western analysis using antibodies against V5-tag, LRRK2, phospho-tau (Thr181), and non-phosphorylated tau. A vector control clone (NEO) expressing only the neomycin gene is shown in lane 1. Right: Graphical representation of the tau (Thr181)-phosphorylation level. (B) Left: WT4-D33 and NEO were treated with 10 µM all-trans retinoic acid for 72 h, transfected with the LRRK2-specific RNAi or RNAi-control, cultured for a further 72 h, and subjected to immunostaining with anti-β III tubulin. Right: Graphical representation of neurite length measured using NIH ImageJ software. Stars represent statistical comparisons by one-way ANOVA (n = 3 in A and n = 50 in B); *: p<0.005, **: p<0.001.

To confirm the tau-phosphorylation activity of LRRK2 in neural cells, we next analyzed a SH-SY5Y clone, WT4-D33, overexpressing V5-wild-type LRRK2 [25]. Western analysis with an antibody recognizing both endogenous and transfected LRRK2 revealed that LRRK2 protein expression by WT4-D33 was about five-fold higher than that in the vector-control clone, NEO (Fig. 4A). We found that this clone exhibited 1.7-fold higher tau (Thr181) phosphorylation than the vector-control. Transfection of LRRK2-specific RNAi into WT4-D33 reduced the level of LRRK2 protein to 46% in comparison with the use of an RNAi control. Concordantly, knockdown of LRRK2 resulted in a significant reduction of tau phosphorylation (Thr181) (76% in comparison with that of the RNAi control cells) (Fig. 4A). Thus, the phosphorylation level of tau (Thr181) was increased or reduced depending on the level of LRRK2 protein *in vivo*, suggesting that tau is likely the physiological substrate for LRRK2.

It has been reported that increase of tau phosphorylation at Thr181 correlate with neurite retraction in SH-SY5Y cells [30], [31]. Therefore, LRRK2 may reduce the neurite length through phosphorylation of tau at Thr181. To analyze neurite outgrowth, WT4-D33 and NEO were first treated with retinoic acid to induce differentiation, and then transfected with the LRRK2-specific RNAi or RNAi-control. The cells were immunostained with anti-beta III tubulin, and then the length of neurites was measured. In the cells transfected with the RNAi-control, WT4-D33 showed significantly shorter neurites than NEO. Conversely, the neurite length of each clone was increased by transfection with the LRRK2-specific siRNA (Fig. 4B). These results indicate that neurite outgrowth is influenced by the level of LRRK2 protein, and together with the above findings, suggest that phosphorylation of tau (Thr181) by LRRK2 is one of the important regulatory mechanisms for neurite outgrowth.

### Effect of LRRK2-mediated phosphorylation of tau on its association with tubulin

**Figure 5:**
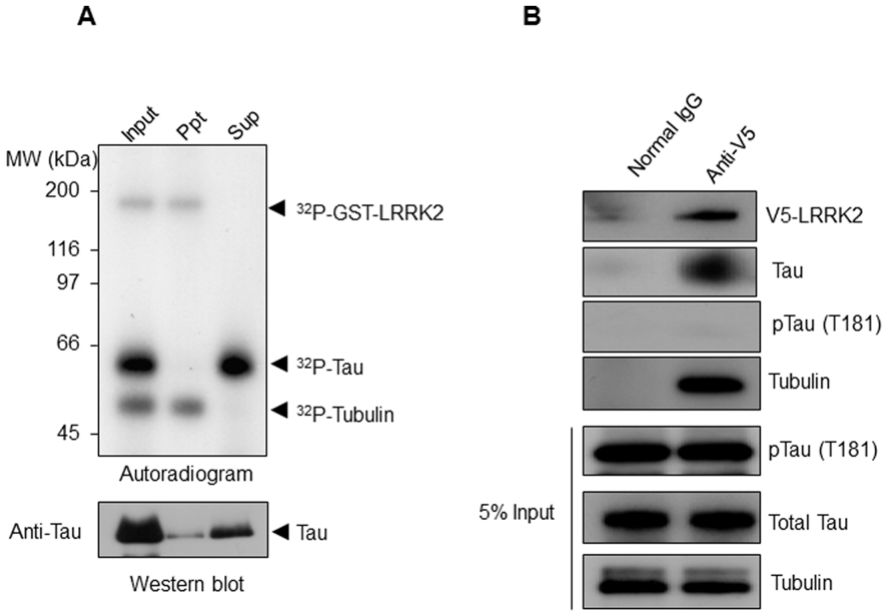
Effect of LRRK2-mediated phosphorylation on the ability of tau to bind to tubulin. (A) Recombinant tau (2 µg) was incubated with GST-LRRK2 (50 ng) and 3 µCi of [γ-^32^P]ATP in the presence of porcine tubulin (1 µg) at 30°C for 120 min and then a GST pull-down assay was performed as described in Materials and Methods. ^32^P-Labeled proteins in the precipitates were detected by autoradiography following SDS-PAGE (upper panel). Total tau in the same sample was detected by Western blotting analysis using anti-tau antibody (lower panel). (B) V5-LRRK2 was immunoprecipitated with anti-V5-agarose beads from a WT4-D33 cell lysate. Precipitated proteins were analyzed by Western blotting using antibodies against V5-tag, phospho-tau (Thr181), non-phospho-epitopes of tau, and tubulin.

As the association of tau with tubulin is well known to be modulated in a phosphorylation-dependent manner, we investigated the effect of LRRK2-mediated phosphorylation on the tubulin-binding capacity of tau. After full phosphorylation of recombinant tau by GST-LRRK2 in the presence of tubulin, we performed a GST pull-down assay. As shown in Fig. 5A, ^32^P-phosphorylated tau was not detected in the precipitated fraction (upper panel), although non-phosphorylated tau was detectable in this fraction (lower panel). In contrast, ^32^P-phosphorylated tau was detected only in the LRRK2-unbound supernatant. Furthermore, we investigated whether tau phosphorylated at Thr181 interacts with LRRK2 using a co-immunoprecipitation assay with a WT4-D33 cell lysate. We found that phosphorylated tau (Thr181) was not detected in the fraction containing LRRK2, whereas immunoreactivity for phospho-independent tau and tubulin was detected in the same fraction (Fig. 5B). These results suggested that LRRK2-mediated phosphorylation of tau abolished its capacity to associate with tubulin. On the other hand, binding of tubulin to LRRK2 was not affected by phosphorylation.

## Discussion

**Figure 6:**
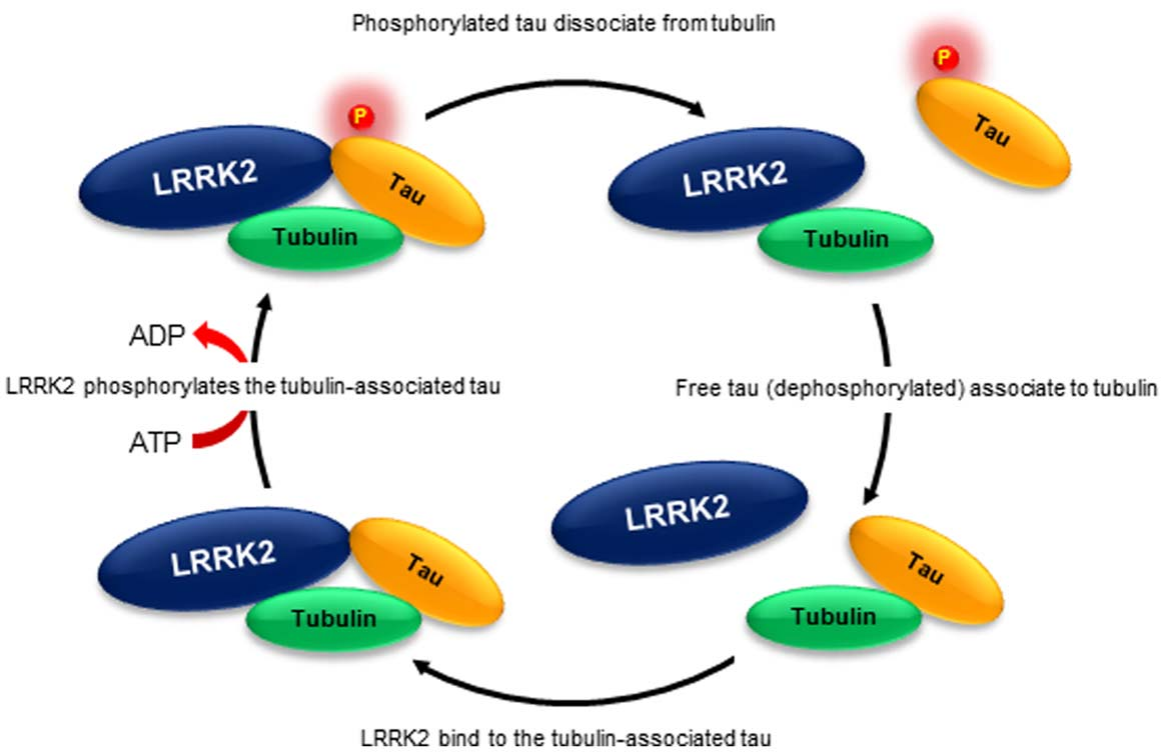
LRRK2-mediated regulation of association/dissociation between tau and tubulin. LRRK2 interacts with tubulin-associated tau, resulting in the formation of a tripartite complex (lower left). This complex induces the phosphorylation of tau by LRRK2 (upper left) and sequentially induces dissociation of tau from tubulin (upper right). Tau is dephosphorylated by certain protein phosphatases, and the dephosphorylated tau then recovers its ability to bind with tubulin (lower right).

In the present study, we found for the first time that LRRK2 directly phosphorylates tubulin-associated tau and reduces its tubulin-binding ability, whereas LRRK2 does not phosphorylate the free tau molecule. Thus, LRRK2 would serve as a regulator of association/dissociation between tau and tubulin (Fig. 6). In neuronal cells, phosphorylation-dependent dissociation of tau from tubulin is an integral aspect of microtubule dynamics essential for neurite outgrowth and axonal transport, although excessive phosphorylation of tau would negatively regulate its ability to promote microtubule assembly. Therefore, LRRK2 may play an important role in neuronal cell function. Importantly, the PD-associated LRRK2 mutations, G2019S and I2020T, were found to exert hyper-phosphorylation of tau, thus providing a clue for clarifying the mechanism of neurodegeneration.

Tau has not been reportedly detected as the LRRK2 binding protein in other studies involving proteomic analysis [32], [33]. Because LRRK2 appears to bind to a wide variety of proteins, including abundant proteins such as chaperones and actin-related molecules, it is possible that the relative proportion of tau among total LRRK2-associated proteins was insufficient for detection in some cases. In the present study, we focused our analysis on tau only, and detected it by Western analysis using a specific antibody. In addition, as we describe here, LRRK2 binds only to tubulin-associated tau and not to free tau, making it more difficult to be detected as a specifically LRRK2-associated molecule. Finally, the LRRK2-overexpressing SH-SY5Y clone from which we prepared the cell lysate for immunoprecipitation was the neuronal cell line known to express a higher level of tau than cells of other tissue types [34].

In the present study, we identified a Thr181 as one of the direct target sites of tau for LRRK2. It has been shown in mouse models that the level of tau phosphorylation at Ser202/Thr205 in the brain was elevated in R1441G and G2019S LRRK2 transgenic mice and diminished in LRRK2-knockout mice [19]–[21]. However, we did not detect LRRK2 phosphorylation of tau at any Ser-residues or at Thr205, nor did we observe any increase in the phosphorylation level of tau Thr205 upon overexpression of LRRK2 in SH-SY5Y cells (data not shown). In a Drosophila model, on the other hand, it has been reported that dopaminergic neurons of G2019S LRRK2-transgenic flies exhibited hyperphosphorylation of tau at Thr212, which was ascribed to kination by the activated GSK-3β homologue, and not to direct kination by LRRK2 [23]. No increase of phosphorylation at either Thr181 or Thr205 was detected in the transgenic flies. These differences in the findings of previous studies could be due either to differences in species, cell sources, or other experimental conditions such as the levels of expression of LRRK2 and tau. LRRK2 has been reported to phosphorylate TAOK3, a kinase with high sequence homology to MARK kinase, suggesting a possibility of LRRK2-mediated indirect phosphorylation of tau [24].

The present results indicating that LRRK2-mediated phosphorylation of tau enhances its dissociation from tubulin suggest that this process is one of the important regulatory mechanisms for microtubule disassembly, which may lead to reduced neurite outgrowth. In mouse neurons, however, there has been some controversy as to whether kinase activity of LRRK2 reduces or promotes neurite outgrowth. Neurite length and branching are reportedly increased by LRRK2-knockdown or LRRK2-kinase inactivation [15], whereas another study has found a decrease of neurons differentiated from LRRK2-knockout mouse embryonic stem cells [14]. Furthermore, kinase active mutant G2019S-LRRK2 expression in neurons has been reported to markedly reduced neurite length in comparison with the wild-type and or kinase-dead mutant [16]–[19].

Several candidate LRRK2-substrate molecules and various mechanisms of neurodegeneration caused by LRRK2 mutations have been reported [8], [35], i.e., the actin-cytoskeleton-related ERM (ezrin/radxin/moesin) whose inappropriate phosphorylation causes perturbation of cytoskeletal organization [36], eukaryotic initiation factor 4E-binding protein 1 (4E-BP1) whose hyperphosphorylation induces dysregulated protein translation [37], and several signal transduction molecules such as TAOK3, serine/threonine kinases 3, 24, and 25 [24], Akt1 [27], and mitogen-activated kinase kinases 3, 4, 6, and 7 [38], whose hypo- or hyperphosphorylation leads to abnormal signal transduction that can induce a wide variety of cellular damage, including activation of the caspase cascade [24], [27], [36]–[40]. With regard to microtubule-related molecules, Gillardon has identified β-tubulin as the LRRK2 substrate and reported that its increased phosphorylation by G2019S LRRK2 enhanced microtubule assembly/stability in the presence of microtubules-associated proteins [14]. Lin et al. have reported that G2019S LRRK2-transgenic Drosophila neurons exhibit GSK-3β-mediated hyperphosphorylation and mislocalization of tau [23]. The R1441G and G2019S LRRK2 transgenic mice exhibiting abnormality in dopamine transmission reportedly have the hyperphosphorylated tau in their brain [20], [21]. The results obtained in the present study demonstrating that G2019S and I2020T mutant LRRK2 elicit direct hyperphosphorylation of tau may further support the notion that impairment of microtubule dynamics plays a crucial role in the neurodegeneration caused by LRRK2 mutations. In addition, LRRK2 immunoreactivity has been detected in tau-positive inclusions in samples of brain tissue affected by various neurodegenerative disorders [41]. In particular, LRRK2 is closely associated with tau-positive inclusions in FTDP-17 caused by N279K tau mutations [42]. It is possible that the LRRK2-mediated phosphorylation of tau reported here may make an important contribution to the formation of these pathological features.
